# CNViz: An R/Shiny Application for Interactive Copy Number Variant Visualization in Cancer

**DOI:** 10.1016/j.jpi.2022.100089

**Published:** 2022-02-15

**Authors:** Rebecca G. Ramesh, Ashkan Bigdeli, Chase Rushton, Jason N. Rosenbaum

**Affiliations:** a Center for Personalized Diagnostics, University of Pennsylvania, Philadelphia, Pennsylvania, USA; b Department of Pathology, Kaiser Permanente, Oakland, California, USA

## Abstract

Copy number variants (CNVs) comprise a class of mutation which includes deletion, duplication, or amplification events that range in size from smaller than a single-gene or exon, to the size of a full chromosome. These changes can affect gene expression levels and are thus implicated in disease, including cancer. Although a variety of tools and methodologies exist to detect CNVs using data from massively parallel sequencing (also referred to as next-generation sequencing), it can be difficult to appreciate the copy number profile in a list format or as a static image. CNViz is a freely accessible R/Bioconductor package that launches an interactive R/Shiny visualization tool to facilitate review of copy number data. As inputs, it requires genomic locations and corresponding copy number ratios for probe, gene, and/or segment-level data. If supplied, loss of heterozygosity (LOH), focal variant data [single nucleotide variants (SNVs) and small insertions and deletions (indels)], and metadata (e.g., specimen purity and ploidy) can also be incorporated into the visualization. The CNViz R/Bioconductor package is an easy-to-use tool built with the intent of encouraging visualization and exploration of copy number variation. CNViz can be used in a clinical setting as well as for research to study patterns in human cancers more broadly. The intuitive interface allows users to visualize the copy number profile of a specimen, dynamically change resolution to explore gene and probe-level copy number changes, and simultaneously integrate LOH, SNV, and indel findings. CNViz is available for download as an R package via Bioconductor. An example of the application is available at rebeccagreenblatt.shinyapps.io/cnviz_example.

## Background

Copy number variants (CNVs) comprise a class of mutation which includes deletion, duplication, and amplification events that range in size from smaller than a single-gene or exon, to the size of a full chromosome. These changes can affect gene expression levels and are thus implicated in disease, including cancer.^1^^,^^2^ For example, *ERBB2* (*Her2*) amplification is considered prognostic in breast cancer and *CDKN2A* deletion is considered diagnostic and prognostic in glioblastoma.^3^

Identifying amplification and deletion events that are correlated with differential gene expression can lead to the discovery of new genes and disease-related pathways.^4^^,^^5^ As an example, by correlating CNV data with gene expression data, *FAM60A* expression was identified as a key prognostic factor in esophageal cancer, leading to a new biomarker and potential therapeutic target.^6^

Large genomic changes (such as trisomy 21) are still resolved by conventional karyotyping (microscopy).^7^ Select CNVs can be identified directly by standalone, targeted assays [such as Reverse Transcriptase Polymerase Chain Reaction (RT-PCR) or Fluorescence In Situ Hybridization (FISH)], whereas others are often not identified at all for clinical management.^8^^,^^9^ Increasingly, massively parallel sequencing (MPS, also referred to as next generation sequencing or NGS) data are being used to estimate copy number across the whole exome or targeted panels of cancer-related genes.^10^^,^^11^ Computational tools like CNVKit^10^ and PureCN^11^ assess targeted short read sequencing data and infer copy number. With appropriate assay design, this strategy offers the opportunity for more comprehensive assessment than standalone assays, higher resolution than conventional karyotyping, and the integration of parallel data detected or inferred from the sequencing. Although CNV information is extremely valuable, it is complex and can be more difficult to appreciate in list format than other types of genetic or genomic changes, such as single nucleotide variants (SNVs) or small insertions and deletions (indels). Visualization of CNV data is often limited to static scatterplots and diagrams.^10^^,^^11^ While these approaches allow for visualization of large events at the resolution of chromosomes or chromosome bands, gene-level or sub-gene-level information is not easily accessible. Interrogation of gene-level information requires access to the raw data and a level of programming competency.^12^

Well-designed visualization tools empower end-users to appreciate and integrate complex information and discover new associations without requiring programming knowledge or extensive data-analytic skills.^13^ Tools like the UCSC genome browser^14^ and the Integrated-Genomics Viewer (IGV)^15^ allow researchers and clinical practitioners to visually inspect variants in the context of their sequencing data. Visual inspection provides an opportunity to review variants identified by informatics algorithms, assess analytical validity of calls made by the algorithm, and characterize the potential biological (and sometimes clinical) consequence of the variants. The demonstrated utility and widespread adoption of these tools in the context of SNVs strongly implies potential utility for analogous tools designed for the visual inspection of CNVs.

The CNViz R/Bioconductor package,^16^^,^^17^ outlined here, takes probe, gene, and segment-level copy number ratios and launches an interactive Shiny application,^18^^,^^19^ enabling detailed visualization of the copy number profile of a given sample. Shiny is an R package that enables the creation of interactive web applications within the R environment, allowing the developer to take advantage of many open source data manipulation and bioinformatics packages. The resulting web applications can run on local servers and are therefore ideally suited to be used with patient-sensitive data. CNViz can integrate parallel data when available, such as loss of heterozygosity (LOH) data and focal variant data (SNVs and small indels) identified by standard bioinformatic variant callers. CNViz also includes aggregated data from The Cancer Genome Atlas (TCGA) 2018 Pan-Cancer Atlas^20^ studies, so the user can correlate findings with those seen in a variety of common cancer diagnoses. While CNViz was developed for use with targeted sequencing panels, the design makes it applicable to assay technologies from small amplicon sequencing to whole genome sequencing.

As an example, we walk through a case seen at the Center for Personalized Diagnostics at the University of Pennsylvania^21^ using CNViz.

## Methods

The launchCNViz function in the CNViz R package^16^ takes properly formatted input data and launches a Shiny application^18^ that visualizes a sample’s copy number profile. Of note, CNViz is purely a visualization tool and thus no inference is performed; the data that are supplied by the user are displayed in an intuitive format that allows for interactive exploration. CNViz does not identify or display fusion genes or chromosomal rearrangements.

**Fig. 1:**
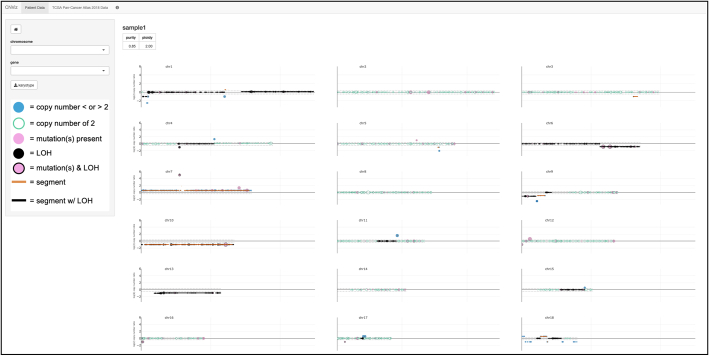
CNViz Homepage. The homepage displays the ‘All Chromosomes’ view. When hovering over a gene marker, the copy number is displayed. If a gene is selected, either by clicking on the marker or by selecting from the dropdown menu on the left, the user will be directed to the ‘Gene View.’

**Fig. 2:**
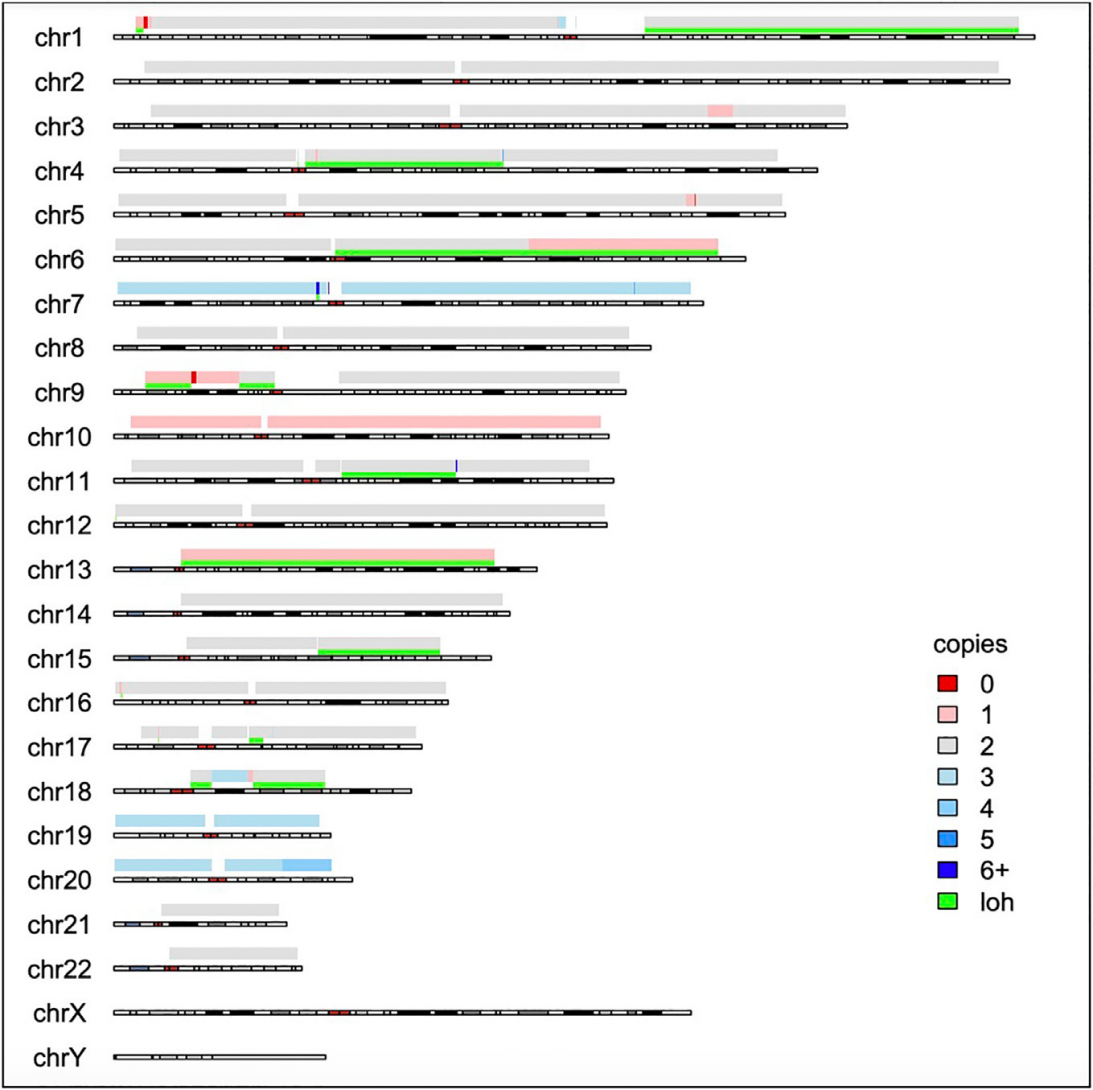
Virtual Karyotype PDF. This image is a reflection of the information displayed in the ‘All Chromosomes’ view. Red indicates loss, blue indicates gain, and green indicates loss of heterozygosity. This karyotype will only be available if segment data is supplied by the user.

**Fig. 3:**
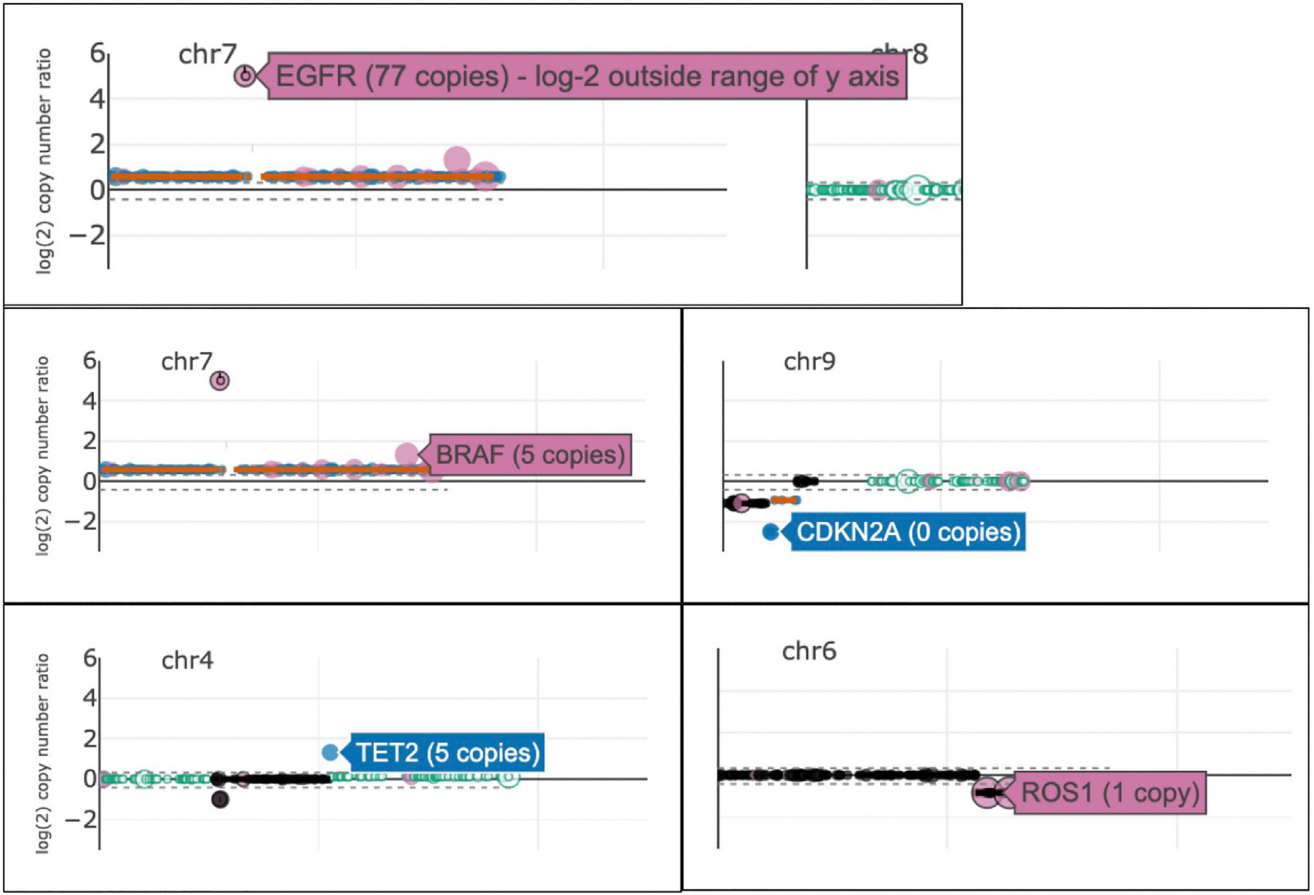
Hover View. From the ‘All Chromosomes’ or ‘Single Chromosome’ view, when hovering over a gene marker, the copy number is displayed. The copy number is rounded to the nearest whole number. If the copy number is out of range (>64 copies), as is seen with *EGFR* in the figure, the correct copy number along with a warning will be displayed. If there are 0 copies, as is seen with *CDKN2A* in the figure, the marker will be displayed at a log-2 copy number ratio corresponding to −2.5.

**Fig. 4:**
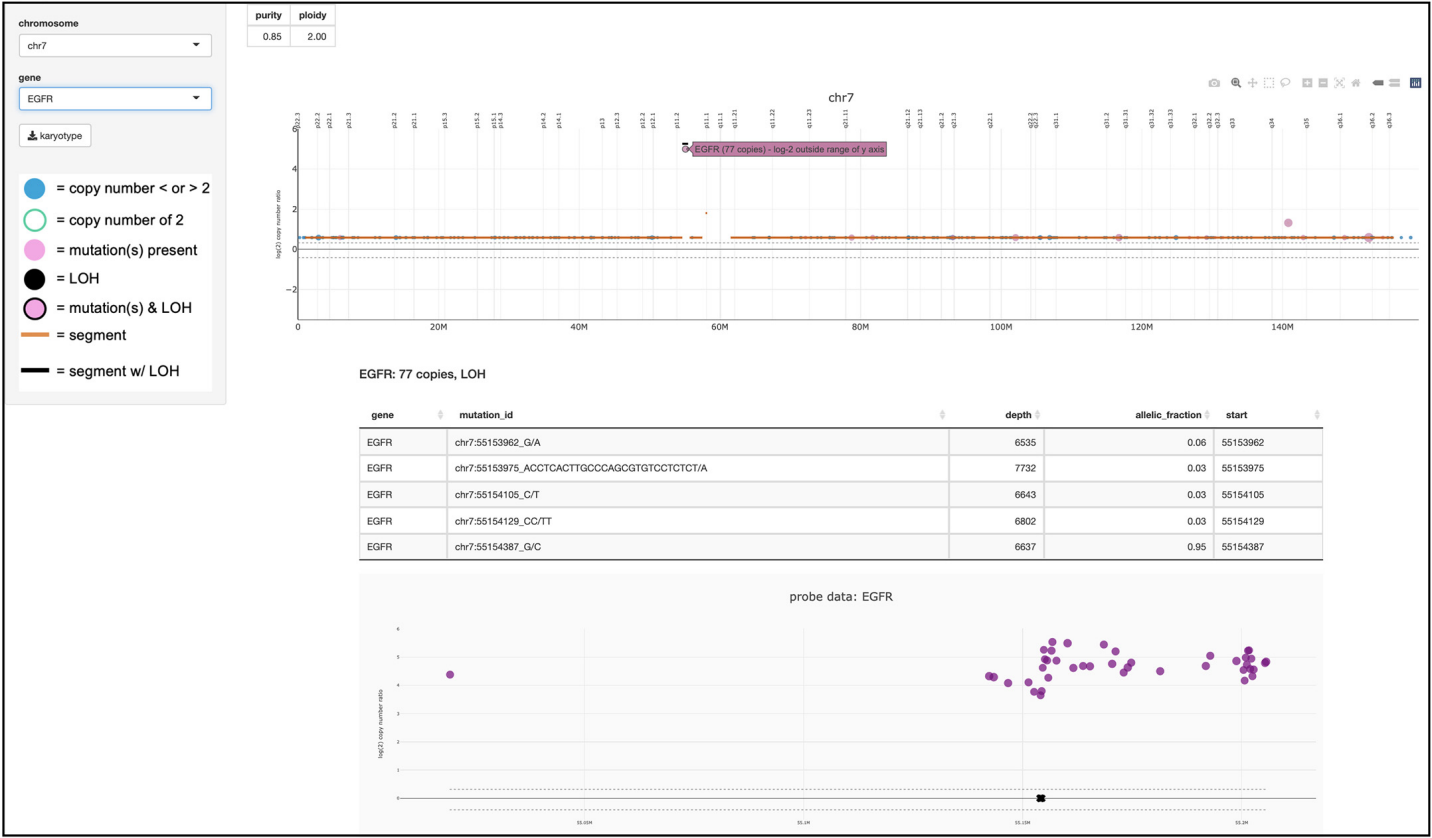
Gene View. When a gene is selected, the user is taken to the relevant ‘Single Chromosome’ view, and gene-specific information is displayed underneath. Specifically, there is a table of focal variants and a plot with probe data. On the probe plot, an “X” on the x-axis indicates that a focal variant is present at that location.

All plots were made using the Plotly R package,^19^ which styles the plots and enables zoom functionality. Design of the application was informed by Shiny and Plotly’s default themes, which are simple and place emphasis on the data represented. The palette was selected with the help of Color Universal Design in order to be colorblind accessible.^22^ The user can view all chromosomes at once (Fig. 1), download a virtual karyotype (Fig. 2), hover to view gene copy number estimates (Fig. 3), view an individual chromosome, and select an individual gene to view probe-level and focal variant data (Fig. 4). These features are all available but will depend on the amount of data supplied to the function. For example, only one of probe-level, gene-level, and segment-level data is required to launch the application, whereas LOH, focal variant, and metadata are optional.

Probe, gene, and segment data inputs are structured similarly. At a minimum, they each must include columns for chromosome, start position, end position, and log-2 copy number ratio. Probe and gene data require the corresponding gene symbol. Genomic coordinates should be given with respect to GRCh38^23^ to ensure the cytoband labels on the x-axis and the karyotype pdf are accurate.

**Fig. 5:**
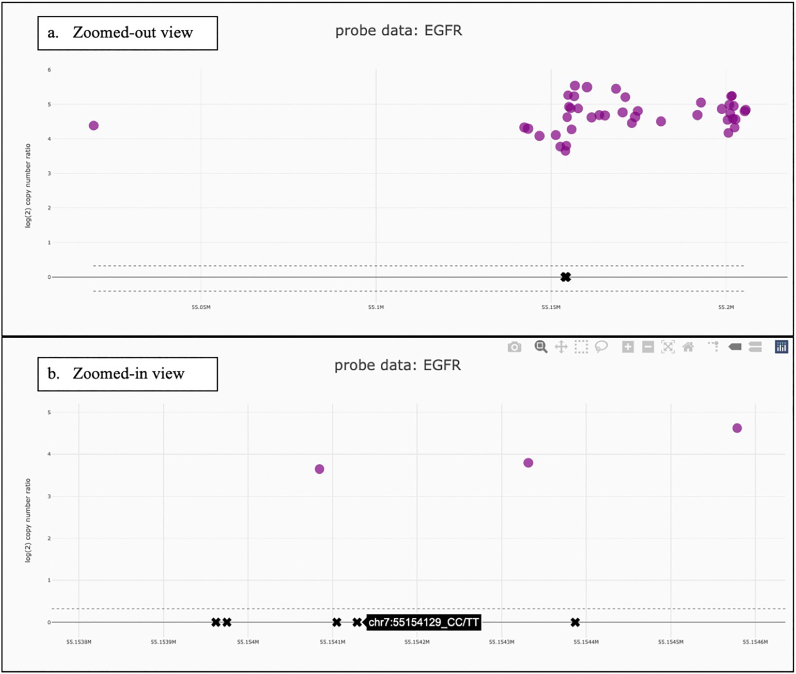
Probe Data. (A) Zoomed-out View. The probe plot of EGFR reveals a possible relative deletion between positions 55.14 and 55.155M. (B) Zoomed-in View. Zooming-in reveals a cluster of variants around 55.154M. Hovering over each “X” displays the corresponding mutation i.d.

LOH can be included as an optional column in gene and segment data and is represented by the gene marker or segment line colored in black. Focal variant data, specifically SNVs and small indels, can be included as well, such that when a gene is clicked, the corresponding variants are displayed in a table below the chromosome plot (Fig. 4) and are plotted on the probe plot (Fig. 5). If included, custom metadata, perhaps the sample’s estimated purity and ploidy, is displayed underneath the sample name (Fig. 1). Ploidy is assumed to be two, unless it is specified in metadata, in which case it is rounded to the nearest whole number. Only segment lines differing from ploidy or with LOH are displayed. All segments from chromosomes X and Y are displayed.

The karyotype PDF is generated using the KaryoploteR,^24^ CopyNumberPlots,^25^ and GenomicRanges^26^ R packages. The PDF will be generated only if segment data is supplied. If the LOH column is included in segment data, then this information will be incorporated into the diagram (Fig. 2).

This application was modeled based on data generated by CNVKit^10^ (probe data) and PureCN^11^ (gene, segment, LOH, focal variant, and metadata), but the aforementioned input data can be obtained through any means, such as a pre-packaged CNV software or a custom-built CNV algorithm. If data include integer copy number (as it does in PureCN), it can be converted to log-2 copy number ratio with log_2_(C/2), where C is the integer copy number. Note that tools like PureCN^11^ adjust integer copy number estimates for inferred purity and ploidy, whereas raw probe-level data might assume a ploidy of 2 and purity of 100%. For this reason, depending on the input data provided, gene and probe log-2 values may not align with one another.

**Fig. 6:**
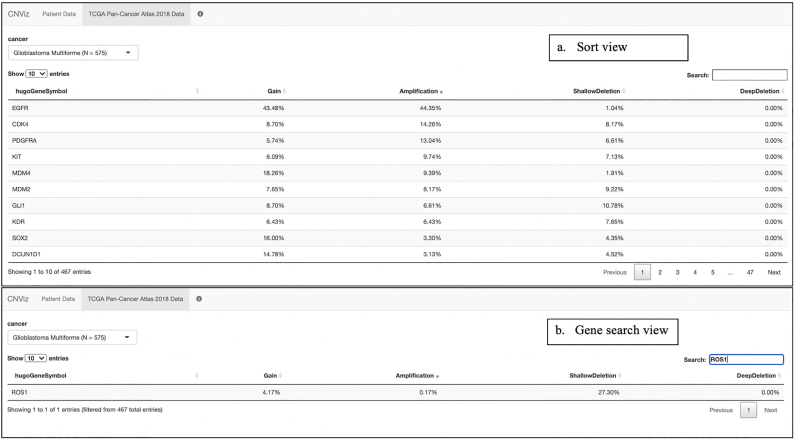
TCGA Pan-Cancer Atlas 2018 Data. The user can select from 32 cancer types. Prevalence of deep deletion, shallow deletion, gain, and amplification for the IMPACT-468 gene panel is displayed. (A) Sort View. The sort feature allows the user to easily identify the most common copy number changes in a specific cancer type. In the figure, the ‘Amplification’ column is sorted, such that the most common amplifications in the Glioblastoma Multiforme cohort are displayed at the top. (B) Gene Search View. The search feature allows the user to see the data for a specific gene of interest.

An additional tab displays data from 2018 TCGA Pan-Cancer Atlas studies (Fig. 6).^20^ These data were obtained through cBioPortal^27^^,^^28^ using the cBioPortalData R package.^29^ The purpose of this tab is to correlate sample findings with aggregated data from the same cancer type. The copy number data displayed were generated by the GISTIC algorithm^30^; they include gain, amplification, shallow deletion, and deep deletion prevalence for 468 cancer-related genes from the IMPACT-468 gene panel.^31^

Shown on rebeccagreenblatt.shinyapps.io/cnviz_example are simulated data; three samples sequenced at the University of Pennsylvania’s Center for Personalized Diagnostics^21^ were modified significantly, then combined to create this example. Specifically, the SNVs were shifted using a random number generator, so the reference nucleotide listed may not line-up with the GRCh38^23^ reference genome. The application was deployed using the rsconnect R package^32^ on the shinyapps.io server^33^ and data are hosted on Amazon S3.^34^

The CNViz R package can be downloaded via Bioconductor^17^ and the code is available at github.com/rebeccagreenblatt/cnviz. The launchCNViz function includes a more detailed description of the required structure of the input data and an example is provided in the corresponding vignette.

## Results

CNViz displays copy number data in an intuitive and interactive format. It allows the user to resolve significant gene-level findings (amplifications, deletions, loss of heterozygosity, SNVs, and indels), as well as integrate these findings into the broader context of the tumor genome (i.e., larger duplication or deletion events). CNViz was modeled based on output from CNVKit^10^ and PureCN,^11^ but can take any input structured in the specified format; at a minimum, the input data must include probe, gene, or segment-level copy number ratios. Any number or combination of genes can be included, such as only the genes included in a predetermined subset, all the genes that were sequenced, or all genes (irrespective of whether they were sequenced or not).

For our example case, the tissue is brain, frontal lobe, and the neoplasm is identified by clinical histology review as glioblastoma multiforme. DNA was extracted from the tissue and sequenced on a clinically validated 152 MPS cancer panel. Probe-level data for 3226 genes was generated using CNVKit.^10^ The PureCN^11^ algorithm estimated gene-level integer copy number, segments, focal variants, and LOH, as well as sample purity and tumor ploidy. The data were transformed minimally in R^16^ to meet the format required for CNViz. For example, column names were standardized, integer copy number estimates were converted to log-2 copy number ratios for gene and segment data, and the LOH column values were simplified to true or false.

After installing the CNViz R/Bioconductor package, the application is launched using the launchCNViz function with the above-mentioned data as input. The homepage loads, which shows all chromosomes (Fig. 1). Clicking on the ‘karyotype’ button downloads a PDF virtual karyotype, which displays gain (blue), loss (red), and LOH (green) segments (Fig. 2). Immediately visualizable are copy-neutral LOH of chromosome 1 and chromosome arm 6p, gain of chromosome 7, and loss of arms 6q, 9p, and chromosome 10. Hovering over gene markers identifies amplification of *EGFR*, gains of *BRAF* and *TET2,* homozygous deletion of *CDKN2A*, and heterozygous deletion of *ROS1*, among other copy number changes (Fig. 3). Also identifiable are focal variants in *EGFR*, *BRAF*, and *ROS1* (indicated by pink markers), and LOH of *EGFR* and *ROS1* (indicated by markers outlined in black).

Clicking on *EGFR* opens the gene view (Fig. 4). This displays chromosome 7 at the top, with *EGFR*-specific information below. For this case, the gene view reveals 77 copies of *EGFR* and suspected LOH. Beneath the gene view is a table of focal variants and a plot of the probe data. The tool permits investigation of parallel or subclonal events, such as the presence of *EGFRvIII*, a variant relevant in tumors of the central nervous system which often co-occurs with *EGFR* amplification.^35^ Visual inspection of the probe plot, reveals a possible relative deletion between positions 55.14M and 55.155M (Fig. 5a). Additionally, the tool displays a cluster of variants around 55.154M, which the tool can further magnify. Hovering over each “X” displays the corresponding mutation identification information (Fig. 5b). Collectively, the amplification, relative deletion, SNVs, and small indels can all be identified and analyzed individually, but also with respect to one another, permitting these variants to be appreciated in their genomic context.

Clicking on the TCGA Pan-Cancer Atlas 2018 Data tab enables comparison of these findings to other publicly available glioblastoma multiforme cases. Selection of glioblastoma multiforme reveals 575 cases in the TCGA 2018 cohort. Sorting by most common amplifications, shows *EGFR* amplification is present in 44.35% of this cohort (Fig. 6a). Searching for *ROS1* shows that 27.3% of cases in the TCGA Pan-Cancer Atlas 2018 cohort had a heterozygous deletion (Fig. 6b).

**Fig. 7:**
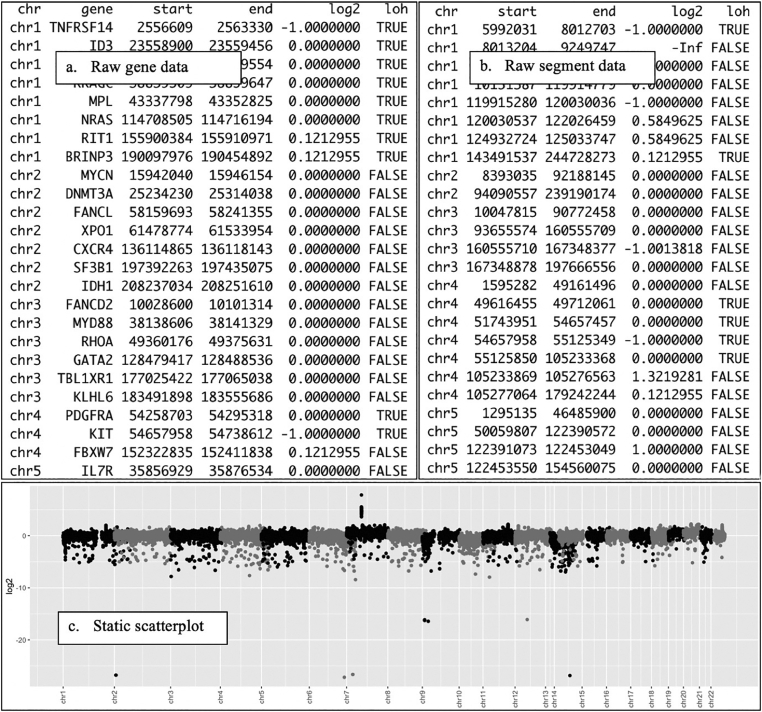
Static Comparison. These figures demonstrate the difficulty of appreciating copy number data in tabulated and static form. (A) Raw Gene Data. While individual gene data can be appreciated, only so many genes can be viewed at once, and it is difficult to integrate individual gene findings into the overall copy number profile of the specimen. (B) Raw Segment Data. Raw segment data does not provide the opportunity to identify which genes fall in each segment. (C) Static Scatterplot. While chromosome-level events can be visualized with a static scatterplot, probe and gene-level data cannot be appreciated.

This workflow may be repeated for any other gene or finding of interest. Analysis and interpretation of CNV data are greatly facilitated by this visualization tool. By comparison, review of a tab-delimited file of gene or segment data (Figs 7a, 7b), makes it much more difficult to appreciate and integrate this information. Static scatterplots (Fig. 7c) are useful for identifying large chromosomal changes, but not for deeper analysis of gene and probe-level data. CNViz enables a more extensive and holistic case review, potentially reducing errors, overlooked data, and increasing the opportunity for integrating findings.

Although there are previously published tools that are improvements on the aforementioned static visualizations, CNViz has a number of attributes that we believe make it a novel contribution to the landscape. For example, while IGV^15^ displays copy number data, it provides only segment-level information. Similar to CNViz, CNspector^36^ and reconCNV^37^ both provide interactive visualization of probe-level copy number data, however, CNViz displays the data in a nested format enabling the user to navigate between chromosome, gene and probe-level views with a single click. This aspect makes the application less visually overwhelming, enabling the user to more immediately hone-in on key gene copy number changes. With CNViz, users can easily click on or search for genes of interest to view more detailed information including probe data, suspected LOH status, and SNVs. Additionally, CNViz displays sample metadata (e.g., evaluated purity and estimated ploidy) as well as provides TCGA data for comparison; this allows reviewers to aggregate meaningful data to accurately assess validity of a putative copy number event.

Future versions of the application will integrate functionality to visualize chromosomal rearrangements and gene fusions, the ability to view variant allele fraction plots as a complement to LOH status, additional cohorts for data comparison beyond the TCGA 2018 data, and information on the level of evidence associated with a copy number event.

A working example of CNViz is hosted on rebeccagreenblatt.shinyapps.io/cnviz_example.^32^ Here, the interactive nature of the application and zoom functionality can be appreciated. In this example, only genes from the University of Pennsylvania’s Center for Personalized Diagnostics^21^ hematologic malignancies panel are displayed, but any number and combination of genes can be included in the visualization.

## Conclusion

The CNViz R/Bioconductor package is a freely accessible, easy-to-use tool built with the intent of encouraging visualization and exploration of copy number variation. CNViz can be used in a clinical setting as well as for research to study patterns in human cancers more broadly. The user-friendly interface allows users to visualize the copy number profile of a specimen, zoom-in to explore gene and probe-level copy number changes, and simultaneously integrate LOH and focal variant data. Furthermore, users can compare findings to those seen in a cohort of patients with a similar diagnosis. Ideally, CNViz will facilitate the identification of clinically meaningful copy number changes in cancer.
