# Erratum to “CNViz: An R/Shiny application for interactive copy number variant visualization in cancer” [Journal of Pathology Informatics Volume 13, 2022, 100089]

**DOI:** 10.1016/j.jpi.2025.100431

**Published:** 2025-03-05

**Authors:** Rebecca G. Ramesh, Ashkan Bigdeli, Chase Rushton, Jason N. Rosenbaum

**Affiliations:** a Center for Personalized Diagnostics, University of Pennsylvania, Philadelphia, PA, USA; b Department of Pathology, Kaiser Permanente, Oakland, CA, USA

The publisher regrets that the Declaration of Competing Interests was missing in the published article. The statement should read as follows:

## Declaration of competing interest

The authors declare that they have no known competing financial interests or personal relationships that could have appeared to influence the work reported in this paper.

The publisher would like to apologise for any inconvenience caused.

